# Editorial

**Published:** 1897-04

**Authors:** 


### Editorial.

### MEDICAL JOURNAL COLLABORATORS.

There seems to be a tendency on the part of every young and aspiring medical journal to attempt to fortify itself behind a list of so-called collaborators, composed of prominent men both here and abroad.

The use of such names, whether sanctioned by their owners or not, has the effect of lending an unmerited weight to an insignificant journal, and is doubtless of value in a business way in securing patronage.

Some years ago a medical journal was launched in Atlanta with an array of co-editors embracing some of the greatest names in medicine of the two hemispheres. It staggered under this burden of greatness and died in three months.

A Western exchange has a list of thirty-four "active collaborators" all well-known medical workers. Some of them with world-wide reputations. Some notion of how active these collaborators are, may be gained from the fact that not a line in the way of original contribution from any except those who live in or near the place of publication has appeared in this journal for over two years, the length of our acquaintance with it.

The collaborators are undoubtedly active, but their activity seems to lie in quite another direction from that of contributing to this particular publication.

The worst of it is that the journal in question (and it is merely one of a large class) is in no need whatever of such quivocal support. It is quite capable of standing alone.

The habit of affixing to their names degrees and distinctions in the form of an inverted triangle with an apex of "etc., etc.," repeated as long as the mechanical requirements demand, which has been popular with medical journal contributors for some time, is dying out. It is overworked and has ceased to be an effective grand-stand play. The "collaborator" business is the same sort of mild fake and can be dispensed with, with the comforting assurance that "it never will be missed."

### MEDICAL ASSOCIATION OF GEORGIA.

The following is the official programme of the State Medical Association. The list of papers is large and interesting, and there is promise of a large meeting:

### PROGRAMME

### OF THE FORTY-EIGHTH ANNUAL SESSION OF THE MEDICAL ASSOCIATION OF GEORGIA, TO BE HELD IN MACON, GEORGIA, APRIL 21, 22, AND 23, 1897.

No member shall be allowed more than twenty minutes in which to read a paper; discussion by any member not to exceed five minntes. Members of the Medical Profession are cordially invited to attend.

### OFFICERS FOR 1897.

President, George H. Noble, M. D., Atlanta; Vice-Presidents, J. B. Morgan, M. D., Augusta, R. B. Barron, M. D., Macon; Secretary, R. H. Taylor, M. D., Griffin; Treasurer, E. C. Goodrich, M. D., Augusta; Board of Censors, Chas. Hicks, M. D., Dublin; C. D. Hurt, M. D., Atlanta; H. B. McMaster, M. D., Waynesboro; H. J. Williams, M. D., Macon; Eugene Foster, M. D., Augusta; Chairman Committee of Arrangements, H. J. Williams, M. D., Macon; Chairman Committee on Programme, Louis H. Jones, M. D., Atlanta.

### FIRST DAY.—WEDNESDAY, APRIL 21.

#### MORNING SESSION.—10.00 O'CLOCK.

Prayer.

Address of welcome, by Hon. N. E. Harris and Dr. W. F. Holt; response by Dr. F. M. Ridley.

President's annual address.

Report of committee of arrangements.

Report of committee on programme.

Report of committee on constitution and by-laws.

Filling vacancies on board of censors.

Applications for membership.

1. A study of the refraction of one thousand eyes: C. H. Peete, M. D., Macon.
2. Entero-colitis of infancy: M. A. Clark, M. D., Barnesville.
3. Some unusual cases in abdominal and gynecological work: K. P. Moore, M. D., Macon.
4. The abortive treatment of acute suppurative inguinal adenitis by pressure bandage: Henry R. Slack, M. D., LaGrange.
5. The treatment of cutaneous cases: J. B. Morgan, M. D., Augusta.
6. Puerperal eclampsia, with report of cases: S. Rumble, M. D., Goggansville.
7. Two cases of malignant tumor affecting the breast, and their treatment: M. B. Hutchins, M. D. Atlanta.

#### AFTERNOON SESSION.—2:30 O'CLOCK.

8. Impurities of drinking water: C. D. Hurt, M. D., Atlanta.
9. Expert testimony in criminal cases involving the plea of insanity: J. C. Olmstead, M. D., Atlanta.
10. Prevention of tuberculosis: J. S. Todd, M. D., Atlanta.
11. A case of cystocele complicating labor: V. D. Lockhart, M. D., Maysville.
12. Functional weakness in organically healthy eyes: J. H. Shorter, M. D., Macon.
13. Some amusing instances of nasal reflex: A. G. Hobbs, M. D., Atlanta.
14. Eucaine, with report of case, D. D. Quillan, M. D., Athens.
15. Influenza with different phases and nervous tendencies: W. O'Daniel, M. D., Bullard's.
16. The neuroses of children: Katherine Collins, M. D., Atlanta.
17. Atypical fevers: E. H. Richardson, M. D., Atlanta.

### SECOND DAY.—THURSDAY, APRIL 22.

### MORNING SESSION.—9.00 O'CLOCK.

Reading of minutes.
Applications for membership.
Report of board of censors.
Report of secretary and treasurer.
Reports of special and standing committees.
Appointment of auditing committee.
18. Meddlesome instrumentation in urethral diseases: W. L. Champion, M. D., Atlanta.
19. Relation of the State Board of Medical Examiners to the profession of the State: E .R. Anthony, M. D., Griffin.
20. Some recent surgical cases: Hunter P. Cooper, M. D., Atlanta.
21. Cystitis: F. M. Ridley, M. D., LaGrange.
22. Fractures of skull, with report of cases: W. B. Tate, M. D., Tate.
23. Physical signs in diseases of the lungs: Louis H. Jones, M. D., Atlanta.
24. Remarks on certain ocular disturbances of general interest: A. W. Stirling, M. D., Atlanta.
25. Rapid dilatation of the uterus, a conservative operation: R. M. Harbin, M. D., Rome.
26. Remarks on fistula-in-ano: W. S. Goldsmith, M. D., Atlanta.

### AFTERNOON SESSION—2.30 O'CLOCK.

27. The induction of premature labor in the interest of the mother and the child: E. R. Corson, M. D., Savannah.
28. Obstetric surgery: Jos. Eve Allen, M. D., Augusta.
29. Ascites in abdominal surgery: Virgil O. Hardon, M. D., Atlanta.
30. The treatment of pleuritis: Newt Anderson, M. D., Eudora.
31. An Act to create a Commissioner of Health and Drainage for the State of Georgia: J. C. LeHardy, M. D., Savannah.
32. Laparo-Hysterotomy, report of a successful case, and a plea for its more frequent adoption: A. Moody Burt, M. D., Sparta.
33. Hernia: W. F. Westmoreland, M. D., Atlanta.
34. Puerperal septicemia, its causes and treatment: J. D. Chason, M. D Iron City.
35. Puerperal infection, its causes and treatment: I. H. Goss, M. D., Athens.
36. The present status of puerperal infection: R. R. Kime, M. D., Atlanta.

### THIRD DAY.—FRIDAY, APRIL 23.

### MORNING SESSION.—9.30 O'CLOCK.

Reading of minutes.
Applications for membership.
Report of board of censors.
37. Spinal paralysis, W. J. Cox, M. D., Barnesville.
38. Some practical suggestions in regard to the tongue, pulse and temperature: W. S. Kendrick, M. D., Atlanta.
39. Some interesting ophthalmological cases: J. Lawton Hiers, M. D., Savannah.
40. Morphine and its effects: Addison K. Bell, M. D., Madison.
41. Some remarks on the treatment of chronic otorrhea: Dunbar Roy, M. D., Atlanta.
42. Keratitis parenchymatosa, a review of seven cases: T. E. Mitchell, M. D., Columbus.
Election of officers at eleven o'clock.
43. Report of case of extra-uterine pregnancy: J. R. Shannon, M. D., Cabaniss.

44. Report of case of empyema in infant, age 17 months, operation, recovery: T. R. Garlington, M. D., Rome.
45. Fractures of the skull, with report of cases: W. S. Elkin, M. D., Atlanta.

### AFTERNOON SESSION.—2.30 O'CLOCK.

46. A successful rhinoplastic operation for extensive destruction of nose. Removal of ossicles for chronic suppurative aural catarrh, cure: J. M. Crawford, M. D., Atlanta.
47. Chorea: Hugh Hagan, M. D., Atlanta.
48. Report of case of central laceration of perineum during labor: P. R. Cortelyou, M. D., Marietta.
49. Lupus of the nose, its medical and surgical treatment: George Horine, M. D., Americus.
50. Dermoid cyst of the bladder with report of case: W. A. Crow, M. D., Atlanta.
51. Hemorrhagic fever: L. P. Hudson, M. D., Cochran.
52. Acranial monster, specimen and report of case: W. P. Williams, M. D., Blackshear.
53. Quinine blindness: A. W. Calhoun, M. D., Atlanta.
54. Some wash drawings illustrating case: George H. Noble, M. D., Atlanta.
55. Infant mortality: E. Van Goidtsnoven, M. D., Atlanta.
56. La grippe: W. C. Lyle, M. D., Augusta.
57. History of epidemics: Marcus F. Carson, M. D., Griffin.

Dr. Jos. Price of Philadelphia, will read a paper.

Dr. Samuel Lloyd, of New York, will read a paper on demonstration of Roentgen rays.

Dr. Hunter McGuire, of Richmond, will read a paper on Appendicitis.

Dr. W. E. B. Davis, of Birmingham, will read a paper on report of cases of pyosalpynx treated by incision and drainage.

### GENERAL INFORMATION.

### PLACE OF MEETING.

The meetings of the Association will be held in the Academy of Music on Mulberry street.

### HOTELS.

The Hotel Lanier. Mulberry street.
The Brown House, Fourth street.
The New Park Hotel, First street.
Will make special rates to members of the Association.

### RAILROADS.

Those attending the meeting must obtain from the ticket agent at point of starting a certificate that they have paid full fare to Macon. These certificates must be signed by the Secretary of the Association at Macon and countersigned by W. P. Dawson, special passenger agent, and upon presentation of these certificates at railroad station in Macon a return ticket will be issued at one cent a mile. Conductors are not allowed to recognize these certificates.

### ENTERTAINMENTS.

The Macon Medical Society will tender the members of the Association a reception at the Cherokee Club on Wednesday evening, April 21st, at nine o'clock, and a barbecue at Ocmulgee Park on Thursday afternoon April 22d, at half past six o'clock.

